# Long-term outcome and prognosis of dissociative disorder with onset in childhood or adolescence

**DOI:** 10.1186/1753-2000-2-19

**Published:** 2008-07-23

**Authors:** Thomas Jans, Stefanie Schneck-Seif, Tobias Weigand, Wolfgang Schneider, Heiner Ellgring, Christoph Wewetzer, Andreas Warnke

**Affiliations:** 1 University of Wuerzburg, University Hospital, Department of Child and Adolescent Psychiatry, Psychosomatics and Psychotherapy, Fuechsleinstr.15, D-97080 Wuerzburg, Germany; 2 University of Wuerzburg, Department of Psychology – Educational Psychology, Roentgenring 10, D-97070 Wuerzburg, Germany; 3 University of Wuerzburg, Department of Psychology – Psychological Intervention, Behavior Analysis and Regulation of Behavior, Marcusstraße 9-11, D-97070 Wuerzburg, Germany; 4 Municipal Hospitals of Cologne, Clinic for Child and Adolescent Psychiatry and Psychotherapy, Florentine-Eichler-Str. 1, D-51067 Koeln, Germany

## Abstract

**Background:**

In the majority of cases short-term treatment outcome of juvenile dissociative disorder is rather favourable. In contrast, the long-term course seems to be less positive, but meaningful results are still fragmentary. The aim of this follow-up study is to bridge this gap to some extent describing the long-term outcome of juvenile dissociative disorder in a clinical sample. To our knowledge there is no comparable other long-term follow-up study which is based on a case definition according to actual classification systems using standardized interviews for individual assessment of the patients at the time of follow-up.

**Methods:**

The total study group was made up of all patients treated for dissociative disorder at our department for child and adolescent psychiatry between 1983 and 1992 (*N *= 62). Two of these former patients committed suicide during the follow-up period (3%). We got information on the clinical course of 27 former patients (44%). 17 out of these 27 former patients were female (63%). The mean age of onset of dissociative disorder was11.7 years and the mean follow-up time was 12.4 years. Most of the patients were reassessed personally (n = 23) at a mean age of 24.8 years using structured interviews covering dissociative disorders, other Axis I disorders and personality disorders (Heidelberg Dissociation Inventory HDI; Expert System for Diagnosing Mental Disorders, DIA-X; Structured Clinical Interview for DSM-IV, SCID-II). Social adjustment was assessed by a semi-structured interview and by patient self report (Social Adjustment Scale – Self Report, SAS-SR). Psychosocial outcome variables were additionally assessed in 36 healthy controls (67% female, mean age = 22.9 years).

**Results:**

At the time of follow-up investigation 82.6% of the patients met the criteria for some form of psychiatric disorder, while 26.1% were still suffering from dissociative disorder. A total of 56.5% presented with an Axis I disorder (especially anxiety, dissociative and somatoform disorders). Personality disorders were seen in 47.8% (especially borderline, obsessive-compulsive and negativistic personality disorders). More dissociative symptoms and inpatient treatment in childhood or adolescence were significantly related to a lower level of psychosocial adjustment in adulthood.

**Conclusion:**

Treatment strategies have to consider that in a significant portion of young patients initial recovery may not be stable over time. Limitations of the study refer to the small sample size and the low rate of former patients taking part in the follow-up investigation.

## Background

Dissociative disorders are characterized by various physical or mental complaints that are not consistent with a known somatic disease. Common symptoms include paralysis, seizures, aphonia, loss of sensation, visual disturbances, amnesia, trance, identity confusion and 'possession' states. Patients may be affected by multiple symptoms and symptoms may change over time. There is no evidence that the manifestation of the symptoms is intentionally produced. A relation to psychological stress or difficult personal circumstances is common. Attention from others and the avoidance of situations difficult to cope with are possible reinforcing conditions.

Childhood dissociative disorder is rarely seen before the age of five [1]. In comparison to adolescents and adults, in children gender ratio seems to be balanced [2-5]. From childhood to adolescence the proportion of affected females as well as prevalence and incidence rates increase [6]. In Australia the incidence of conversion disorder in specialist child health practices was estimated to be about 4/100 000 [7]. In a large German epidemiologic sample of adolescents and young adults aged between 14 to 24 years lifetime prevalence was 0.4% for conversion disorder and 0.8% for dissociative disorder not otherwise specified [8]. With respect to samples of child and adolescent psychiatric care rates vary considerably (0.5% to 17%) [9]. Apart from differences in case definition and different structures of clinical referral this variation may also be due to cultural influences [10].

In general, treatment outcome of juvenile dissociative disorder is rather favourable. Rates of patients with complete remission or significant improvement at the end of treatment or at short-term follow-up (≤ 6 months) vary between 44% and 97% [9,11,4,10,5,16]. However, at middle-term or long-term follow up the rate of patients with dissociative symptoms or disorder ranged from 14% to 55% [[17,1,14,3-25]; a review in German: [26]]. Co-morbidity rates varied between 12% and 38%. Anxiety and depressive disorders were diagnosed most frequently. Thus middle-term and long-term course seems to be less positive in comparison to remission rates reported after initial treatment in childhood or adolescence. However, meaningful results on the long-term course are fragmentary. To our knowledge there are only three studies reporting a mean follow-up period exceeding five years [17,21,24]. There is no study that presents results based on a case definition according to actual classification systems using standardized interviews for individual assessment of the patients at the time of follow-up. Studies investigating the development of personality disorders and the psychosocial functioning in adulthood are missing as well.

The aim of the present follow-up study was to describe the long-term outcome of juvenile dissociative disorder and to assess psychiatric morbidity including personality disorders in the follow-up patients. Furthermore, we assessed different areas of psychosocial functioning (occupational functioning and financial situation, social life: relations to the family of origin, friendships and social contacts, partnership and sexuality). With respect to these psychosocial outcome variables we additionally investigated a control group of healthy subjects. Predictors in childhood for the level of psychosocial adaptation in adulthood were examined in the follow-up sample. Due to inconsistent previous studies our study was designed to be exploratory and formal hypotheses were not formulated. Our results will be discussed in the context of previous findings.

## Methods

### Sample

The total study group was made up of all patients who had received treatment for dissociative disorder at the Department of Child and Adolescent Psychiatry of Wuerzburg University between 1983 and 1992. Using a structured checklist, patient charts were reviewed carefully by experienced clinicians and the final study group included only those patients who met the ICD-10 diagnostic criteria for dissociative disorder [27]. Further inclusion criteria were: age at follow-up ≥ 18 years and IQ > 70 (no mental retardation, as measured upon initial referral). All subjects received 50 € for their participation. The Ethics Committee of the University of Wuerzburg granted approval of the study.

A total of 62 patients met these criteria. Despite extensive efforts both by mail and phone, only 23 subjects of the original sample agreed to be interviewed personally. We managed to conduct an interview by telephone with two patients and received information by self-ratings from another two patients. Consequently, we were able to gather information on the clinical course of 27 (44%) of our former patients. Two former patients had committed suicide. 10 patients could not be traced, two lived outside of Germany and 21 patients refused to take part in this investigation.

Characteristics of the follow-up sample are shown in table 1. Two thirds of the patients were female. Most of the patients had no prior psychiatric treatment and were readmitted to inpatient treatment upon initial referral to our department in the majority of cases. The mean age at onset of dissociative disorder was 11.7 years and the mean age at diagnosis of the disorder was 12.6 years, which corresponded to the mean age at the start of treatment for the disorder (12.7 years). The mean length of the follow-up period was 12.4 years. At the time under exploration treatment strategies at our department were predominantly analytical. Psychopharmacological treatment had been sparse. At the end of treatment the dissociative symptoms of 89% of our patients had been rated as recovered or markedly reduced by the therapist.

**Table 1:** Follow-up sample (*n *= 27)

female	63%	
mean age at onset of dissociative disorder	11.7 years (*SD *= 2.54; *min *= 6; *max *= 17)	

mean age at diagnosis of dissociative disorder	12.6 years (*SD *= 2.71; *min *= 8; *max *= 17)	

mean age at follow-up	24.8 years (*SD *= 5.4; *min *= 18; *max *= 36)	

mean length of follow-up period	12.4 years (*SD *= 4.9; *min *= 2; *max *= 19)	

socioeconomic class ^1 ^(according to the professional status of the parents)	low:	56%
	middle:	28%
	upper:	16%

Dissociative symptoms ^1^	anaesthesia and sensory loss:	41%
	motor disorders:	41%
	convulsions:	33%
	amnesia:	11%
	trance and possession disorder:	4%
	multiple dissociative symptoms: (mixed dissociative disorder)	33%

co-existing symptoms ^1^	somatoform symptoms:	30%
	anxiety:	30%
	aggressive behaviour:	22%
	parasuicidal tendencies:	19%
	motor tics:	19%
	depression:	11%
	substance related problems:	11%
	school refusal:	11%

treatment before consulting our department	inpatient:	15%
	outpatient:	7%

treatment at our department	inpatient:	70%
	outpatient:	30%
	predominately analytical:	59%
	predominately family therapy:	41%
	predominately behaviour therapy:	19%
	additional psychopharmacological treatment:	15%
	terminating treatment against medical advice:	5%

treatment outcome at our department	recovered or markedly reduced symptoms:	89%
	no or little improvement:	11%

^1 ^at the time of initial referral at our department.

At the time of the first consultation, most of the patients suffered from anaesthesia and sensory loss, motor disorders or convulsions. Altered sensations were predominantly cutaneous or visual. Disturbed motor control most commonly implied deglutition or resulted in astasia or abasia. Arms were affected less often than legs. Pseudoseizures were mostly presented with fainting, generalized uncoordinated movements, spasm, trembling, staring or oculogyric crisis. Few patients had amnesia or possession disorder. Dissociative fugue, stupor, multiple personality disorder or Ganser's syndrome did not occur. In mixed dissociative disorder patients concurrent motor and sensory symptoms were the most common combination (60% of the patients with multiple symptoms). Commonly associated coexisting features included somatoform symptoms, anxiety and aggressive behaviour.

At the time of treatment at our department during childhood or adolescence the mean IQ of the 27 patients was 102.7 (*SD *= 13.65). Anamnesis refers to a delay in speech or motor development in 8% of the patients. Pathological EEG findings did not occur.

Psychosocial outcome variables were additionally assessed in a control group of 36 subjects (11 males, 33%; mean age = 22.9 years, *SD *= 2.16, *min *= 18, *max *= 29). 32 subjects were students. Since four patients in our study group had below average I.Q., 4 control subjects were recruited from an institution for vocational preparation for mentally handicapped young adults. All control subjects were free from mental disorders ascertained by individual assessment using a semi-structured diagnostic interview (see measures section). None of the controls reported dissociative disorder in childhood.

#### Differences between participants and non-participants

The statistical comparison of the 27 participants with the 35 non-participants revealed an earlier onset of dissociative disorder in the participants (participants: *m *= 11.7, *SD *= 2.54; non-participants: *m *= 13.5, *SD *= 2.73; *df *= 60; *t *= -2.7; *p *< 0.01). Participants also tended to be younger at the time of first treatment for dissociative disorder (participants: *m *= 12.7, *SD *= 2.68; non-participants: *m *= 14.0, *SD *= 2.91; *df *= 60; *t *= -1.9; *p *= 0.07). From these data it can be deduced that the time between onset and treatment of the disorder was longer in participants as compared with non-participants (about 12 months vs. 6 months). With respect to other variables in childhood (severity of dissociative disorder at referral, subtype of dissociative disorder, co-morbid symptoms, inpatient versus outpatient treatment, therapeutic strategies; termination of treatment against medical advice, treatment response, gender, speech or motor development, neurological findings, intelligence, socioeconomic background) the two groups did not differ significantly.

### Measures

A documentation system was developed to allow for a systematic retrospective assessment of data by chart review, which included medical, social and family history, clinical symptomatology and the course of dissociative disorder, psychopathology, therapy and outcome. A diagnosis according to ICD-10 on the basis of the case records was limited to dissociative disorder. The associated psychopathology was described on the level of co-existing symptoms.

At follow-up, psychiatric disorders were diagnosed according to ICD-10 criteria using the Expert System for Diagnosing Mental Disorders (DIA-X) [28,29]. The DIA-X is based on the Composite International Diagnostic Interview (CIDI) [30,31]. Both interviews have a high interrater-reliability and satisfactory test-retest reliability for most diagnostic categories. Their validity has also proven acceptable. The diagnoses referred to in the results relate to the day of the interview and the preceding six months. To allow for a more detailed investigation of dissociative disorders, in addition to the DIA-X the Heidelberg Dissociation Inventory (Heidelberger Dissoziationsinventar, HDI) was applied [32]. The structured interview covers 10 dissociative disorder categories. Point prevalences according to ICD-10 criteria will be described below. Interrater-reliability and validity of the interview have proven sufficient. The Structured Clinical Interview for DSM-IV (SCID-II) was applied for diagnoses of personality disorder [33]. In terms of its reliability and validity, SCID-II compares well with other assessment measures for personality disorders. With respect to Axis I disorders we decided to rely on ICD-10 classification because dissociative disorders are more consistently grouped together here as compared to DSM-IV [34,35]. With respect to personality disorders our decision for DSM-IV classification is based on the strategy used in previous follow-up studies of our research group [36,37]. A major reason for using DSM-IV classification was the earlier availability of diagnostic interviews in German as opposed to ICD-10 (this holds for DSM-III-R) [38].

In addition, a semi-structured interview was administered to assess treatment episodes during the follow-up period, tic disorders and social adjustment. Adapted for the disorder under investigation this interview was used in previous follow-up studies by our research group [37,39]. This interview was supplemented by the German version of the Social Adjustment Scale – Self Report (SAS-SR; Fragebogen zur sozialen Integration, FSI) [40,41] and the Global Assessment of Functioning (GAF; to assure compatibility with our former follow-up studies the version of the scale presented in DSM-III-R using scores ranging from 1 to 90 was applied). GAF scores reflect both the level of psychosocial adjustment and global symptom severity.

The telephone interviews were only structured by a brief checklist and did not include the diagnostic instruments used for the personal investigation. Subjects in the control group were interviewed with the semi-structured interview described above and filled out the FSI to assess psychosocial functioning. The screening for psychiatric morbidity included the M-DIPS (German short version of the Anxiety Disorders Interview Schedule-Revised, ADIS-R) [42,43].

### Reliability test

This study is part of a series of follow-up studies conducted at our and allied departments. The methodology of this investigation is comparable to that of a study on the long-term course of obsessive-compulsive disorder [37]. The same researchers were involved in the training and supervision of the interviewers (AW, CW, TJ). Thus we refer to data on reliability collected during the follow-up study on OCD. Chart review was highly reliable (inter-correlation of ages at onset of OCD: *r *= 0.96; the relation between the corresponding judgements and all judgements given by two investigators varied between 0.86 and 0.96 for symptoms and for the applied therapeutic interventions; corrected for inter-judge agreement expected by chance these values varied between 0.66 and 0.86; Flander's π, according to Friede [44], and Cohen's Kappa were applied). An inter-judge agreement of 100% with respect to CIDI-diagnoses was due to the highly standardized character of the interview and did probably not apply to the other, semi-structured interviews conducted during the follow-up investigation for which no reliability check was measured. However, this high agreement reflects the accuracy of the personal assessment of our patients at the time of follow-up.

### Statistical analysis

Patients of the follow-up group (*n *= 27) and those not re-examined (*n *= 35) were compared in order to evaluate the representativeness of the follow-up sample. At follow-up patients and controls were compared with respect to psychosocial functioning. χ^2^-Test, Fisher's-Exact-Test, phi-coefficient, Mann-Whitney-U-Test or t-Test were used for single comparisons. All p-values are nominal. Pearson's, point-biserial or Spearman's correlations were used to test the associations between the predictors in childhood and GAF-scores in adulthood. Due to the small sample size no multivariate analyses were performed. All analyses were computed using SPSS for Windows 14.0.

## Results

### Clinical disorders (Axis I disorders) and personality disorders

**Figure 1:**
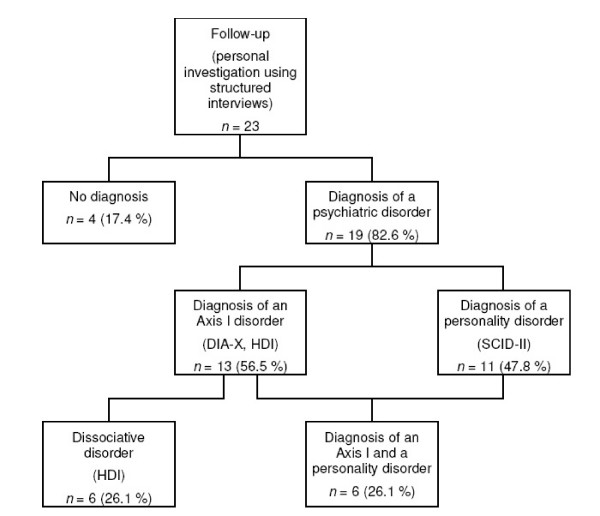
**Psychiatric disorders at the time of the follow-up (*n *= 23)**. DIA-X: Expert System for Diagnosing Mental Disorders; SCID-II: Structured Clinical Interview for DSM-IV; HDI: Heidelberg Dissociation Inventory.

At the time of follow-up a psychiatric disorder was diagnosed in 83% of the 23 personally interviewed patients (see figure 1). Any Axis I disorder – dissociative disorders included – was diagnosed in 57%. 26% still suffered from a dissociative disorder. A personality disorder was seen in 48%. 26% were diagnosed with both, an Axis I and a personality disorder.

With respect to the subtype of dissociative disorder present at the time of follow-up two patients had dissociative amnesia, two patients had dissociative convulsions and two patients had dissociative anaesthesia and sensory loss (8.7% respectively out of 23 personally investigated patients). Among these six patients two were male and four were female. No patient presented with a mixed dissociative disorder. Regarding treatment for dissociative disorder during the entire follow-up period we obtained information from 27 patients. Three out of these 27 former patients (11.1%) had received outpatient treatment after discharge from our clinic, three had inpatient treatment (11.1%), two had both inpatient and outpatient treatment (7.4%) and another three patients reported untreated dissociative disorder (all three of these patients had received an actual diagnosis of dissociative disorder). The patients were asked if an organic disorder had been diagnosed during the follow-up period that accounted for their dissociative symptoms. None of the patients reported an underlying organic cause of the symptoms.

Table 2 gives information on Axis I disorders according to ICD-10 at the time of follow-up. Apart from dissociative disorder anxiety disorders and somatoform disorders had been diagnosed most frequently. Affective disorders were less common. However, two patients out of the original sample of 62 patients committed suicide.

**Table 2:** Axis I disorders according to ICD-10 research criteria at the time of the follow-up using DIA-X and HDI (*n *= 23; in some of the patients more than one disorder was diagnosed)

Any Axis I disorder	13	(56.5%)
Dissociative disorder	6	(26.1%)
Somatoform disorder	5	(21.7%)
Phobic disorder (social or simple phobia)	3	(13%)
Agoraphobia	4	(17.4%)
Dysthymia	3	(13%)
Bipolar disorder	1	(4.3%)
Substance related disorder	2	(8.7%)

DIA-X: Expert System for Diagnosing Mental Disorders; HDI: Heidelberg Dissociation Inventory.

The frequency of personality disorders is shown in table 3. Borderline, obsessive-compulsive, negativistic and avoidant personality disorder were the most frequent disorders.

**Table 3:** Personality disorders according to DSM-IV criteria at the time of the follow-up using SCID-II (*n *= 23; in some of the patients more than one disorder was diagnosed)

Any personality disorder	11	(47.8%)
Borderline	5	(21.7%)
Obsessive-compulsive	4	(17.4%)
Negativistic	3	(13%)
Avoidant	2	(8.7%)
Dependent	1	(4.3%)
Histrionic	1	(4.3%)
Depressive	1	(4.3%)
Paranoid	1	(4.3%)
Schizotypical	1	(4.3%)
Schizoid	1	(4.3%)

SCID-II: Structured Clinical Interview for DSM-IV.

### Psychosocial functioning

Interview data on psychosocial functioning refer to the statements of patients and controls with respect to their family of origin, to friendship and social contacts as well as to partnership and sexuality. Most of the patients and controls reported good family relations with no significant differences between patients and controls (patients: 76%, controls: 80%). However, an age appropriate financial and emotional independence from the parents was found only in 72% of the patients as compared to 94% of controls (*p *< 0.05). In line with this rating more patients than controls reported to still be living together with their parents (50% vs. 14%, *p *< 0.01). At least weekly social leisure activities outside from home had been reported by 70% of the patients and 94% of controls (*p *< 0.05). Although not significant on a statistical level less patients than controls reported being visited at home at least weekly (46% vs. 69%). Less patients than controls reported having very close or close friends (62% vs.100%; *p *< 0.01). Nevertheless, both groups reported on a similar level contentment with their social contacts outside their family of origin (patients: 88%, controls: 91%). With respect to partnership there were no significant differences between patients and controls (42% of the patients and 57% of controls stated to have a partner; out of the subjects with partner 100% of the patients and 95% of the controls reported to be mainly or throughly content with their partnership). However, more directly interviewed regarding sexuality the two groups differed significantly. 71% of the patients reported enjoying sexual activities while 97% of controls reported sexual satisfaction (*p *< 0.05). 31% of the patients stated that they avoided sexual contacts compared to no controls (*p *< 0.05). Less patients than controls indicated having had sexual intercourse (67% vs. 97%, *p *< 0.05). With respect to the questions asked about their sexual life 41% of the patients in comparison to 9% of the control stated at least once of being unable or unwilling to give a rating.

FSI ratings did not differ significantly between control subjects and patients with regard to family relations, partnership, occupational functioning and financial situation. With respect to social contacts and leisure activities (categories: regular activities; irregular activities; minimal or no activities) patients reported significantly more often minimal or no activities as compared to controls (36% vs. 6%; *p *< 0.01). This was especially the case for patients suffering from psychiatric disorders at the time of follow-up as compared to patients without psychiatric disorders (42% vs. 17%).

The global rating on social, occupational and psychological functioning provided by the GAF-scale revealed that 68% of our patients had GAF-scores of 61 or higher (no or some mild symptoms or some difficulty in social, occupational, or school functioning) and 20% had a score between 51 and 60 (moderate symptoms or any moderate difficulty in social, occupational, or school functioning). Only 8% had serious symptoms or any serious impairment and 4% had inability to function in all areas. The mean GAF-score implies a relatively good psychosocial adaptation of the follow-up sample (*m *= 69.6, *SD *= 15.47, *min *= 30, *max *= 90).

### Predictors of the clinical course

Table 4 shows correlations between predictors in childhood and GAF-scores. For most of these variables no associations were found. However, more dissociative symptoms in childhood or adolescence were strongly related to a lower level of psychosocial adjustment at the time of follow-up. Patients treated at our outpatient unit had significantly higher GAF-scores than inpatients. There was a tendency for a lower adjustment in patients with co-morbid anxiety in childhood and adolescence.

**Table 4:** Correlations between predictors in childhood and psychosocial functioning in adulthood (GAF; *n *= 25)

Gender ^2^	*r*_*pbi *_= 0.13
Age at onset of dissociative disorder ^1^	*r *= -0.14
Severity of dissociative symptoms ^1^	*r*_*rho *_= 0.19
Dissociative convulsions ^1^	*r*_*pbi *_= 0.21
**Number of dissociative symptoms **^1^	***r***_*rho *_= -0.65 **
**Co-morbid anxiety **^1^	***r***_*pbi *_= **0.27 +**
Outcome ^1^	*r*_*rho *_= -0.12
**Setting (outpatient/inpatient) **^1^	***r***_*pbi *_= 0.43 *
Terminating treatment against advice ^1^	*r*_*pbi *_= 0.03
Length of follow-up period ^2^	*r *= 0.06

r, r_pbi_, r_rho_: Pearson's, point-biserial, Spearman's correlations, + *p *≤ 0.1, * *p *≤ 0.05, ** *p *≤ 0.01, ^1 ^one-tailed significance, ^2 ^two-tailed significance.

## Discussion

The present study is the first investigating the long-term course of juvenile dissociative disorder with respect not only to dissociative symptomatology but also to Axis I and II disorders and psychosocial functioning at the time of follow-up in adulthood using standardized diagnostic methods. However, only 44% of our former patients could be reassessed. In other long-term follow-up studies higher rates of participants have been reported (90% after 8.6 years [17]; 96% after 12.6 years [21]). On the other hand problems in the recruitment of patients for follow-up also emerged in studies on childhood dissociative disorder even with shorter follow-up periods (51% after 40 months [22]). In a recent 14 months follow-up study on adult dissociative disorder 56% of the patients could be reassessed only [45]. From the detailed comparison of participants and non-participants, in our study no significant differences were found except for a later onset of dissociative disorder in the non-participants. With regard to childhood variables in our study there seems to be no systematic bias due to the low rate of participation.

Our sample fits well with other studies on juvenile dissociative disorder. A high rate of our patients was adolescent (mean age of 12.7 years). In line with this age pattern there were more females than males in our sample. Most of our patients had a favourable treatment response at the end of treatment at our department. The finding that conversion disorders affecting motor control and sensation as well as pseudoseizures were in the foreground is well in line with other studies on child psychiatric and paediatric samples. This holds for the proportion of patients with mixed symptomatology as well [13,44,7]. It should be noted that most of our former patients had symptoms of conversion and would have been classified as having somatoform disorder according to DSM-IV's terminology (in DSM-IV the term "dissociative" only refers to mental and cognitive symptoms and does not include pseudo-neurological symptoms of conversion). In our sample the most frequent co-existing features were somatoform symptoms, anxiety and aggressive behaviour. Other studies also reported on internalizing co-morbidity, pain and externalizing symptoms [11,47,7].

After a mean follow-up period of 12.4 years 26% of our patients still suffered from dissociative disorder. 41% of our patients reported dissociative symptoms requiring treatment for at least one particular time during the follow-up period. In accordance with these results other studies reported rates between14% and 55% of patients with dissociative symptoms or disorder present at middle-term or long-term follow up [26]. In summary, our results also point to the fact that in a significant proportion of patients improvement during initial treatment is not stable over time.

None of our patients reported that a bodily abnormality or clinical somatic diagnosis had been discovered during the follow-up period that accounted for their dissociative symptoms. Even if the accuracy of the patients' statements with respect to differential diagnostic might be challenged, this points to a high validity of the dissociative disorder diagnosis reported otherwise (rates of misdiagnosis between 0% and 17% [26]). In a recent metaanalysis including predominantly adult samples a significant decline in the mean rate of misdiagnosis from the 1950s to present was found (29% in the 1950s; 17% in the 1960s; 4% in the 1970s, 1980s and 1990s) [48]. The authors concluded that this decline was probably due to improvements in study quality rather than improved diagnostic accuracy since it preceded the growing availability of computer tomography in the late 1970s. As a guideline, a well-founded diagnosis of dissociative disorder is always based on positive signs of conversion or dissociation and must not be reduced to the mere absence of physical findings.

In follow-up studies of children and adolescents the reported rates of patients with co-morbid Axis I disorders vary between 12% and 38% ([17]: 38%, [2]: 20%, [21]: 12%, [22]: 32%, [25]: 35%). Anxiety and depressive disorders had been diagnosed most frequently. However, only in the study conducted by Pelivanturk & Unal [25] standardized diagnostic interviews were used. In our sample co-morbidity was present even more frequently: 48% of our patients presented an Axis I disorder (dissociative disorders not included). Apart from the standardized assessment applied this relatively high rate of co-morbidity may be due to the fact that our patients had a mean age of 24.8 years at the time of follow-up. Among adult patients with dissociative disorders Axis I co-morbidity is reported to be pronounced ([49]: another Axis I disorder: 78%, mainly anxiety and depression; [50]: anxiety: 72%, substance related disorders: 46%, depressive episode: 30%, recurrent depression: 40%, somatoform disorders: 20%; [51]: anxiety disorder: 79%, somatoform disorder: 76%, affective disorder: 71%). In our child psychiatric sample we found an increase in somatization symptoms, which is also reported for the clinical course of adult dissociative disorder patients [52-54].

To our knowledge there is no other child psychiatric sample of dissociative disorder patients followed-up with regard to the development of personality disorders. Nearly half of our patients had a personality disorder at the time of follow-up with borderline, obsessive-compulsive, negativistic and avoidant personality disorder being diagnosed most frequently. In adult dissociative movement disorder patients Axis-II co-morbidity was found in 53% of the patients [49]. Dependent, borderline and avoidant disorders had been diagnosed most frequently. Histrionic personality disorder was only seen in 6% of the cases. Personality disorders had also been diagnosed frequently in patients with nonepileptic seizures [55]. The co-occurrence of borderline personality and dissociative disorder is especially common [51,45]. On the other hand in a community based longitudinal study individuals with personality disorders were substantially more likely than those without personality disorders to have had dissociative disorder [56]. Summing up, we found a high rate of Axis-II co-morbidity especially with respect to cluster B and C personality disorders among which histrionic personality disorder was diagnosed rather infrequently.

In contrast to the high psychiatric morbidity at the time of follow-up psychosocial functioning was quite good with no or little impairment in about 70% of our former patients. Impairments had predominantly been related to a higher dependency from parents, to less intense social contacts and a less satisfying sexuality. With respect to psychosocial functioning other follow-up studies reported inconsistent results. The rate of patients with impairments or only minor improvements in at least one area of psychosocial adjustment varied between 10% and 69% [26]. Long-term follow-up pointed to serious impairments in the study by Robins & O'Neal [17]. More positive results were reported by other research groups [20,18,21]. In line with other studies [1,22] we found an association between psychiatric morbidity and psychosocial functioning. However, with respect to the GAF-scores this finding may be partly tautological since GAF-ratings reflect the level of psychosocial adjustment as well as global symptom severity.

With respect to prognostic factors for the course of juvenile dissociative disorder most of the results of existing studies are inconsistent. The comparison of study results is complicated by differences in methodology (e.g. case definition, characteristics of referral to the study centres, diagnostic strategies, length of follow-up period, criteria to be predicted by childhood variables). A better prognosis was found in dissociative disorder children and adolescents with younger age at first presentation [24]. Irwin et al. [23] hypothesized that prognosis of children may be better perhaps because causes are more likely to be external to the child, more easily identified, and more amenable to prompt intervention. However, in contrast others reported a tendency to a more serious course in patients with early onset [22]. Age at onset was not associated with outcome in our study. Inconsistent results on the predictive value had also been reported with regard to gender (no association: three studies [57,22,25] and our study; a more favourable course for females: two studies [17,24]). Initial treatment response or referral to psychiatric care was not associated with the middle-term course [20,57]. However, intensity of treatment may be confounded with the severity of symptoms. Gudmundsson et al. [24] reported that patients not receiving both inpatient and outpatient treatment had a more favourable course. In line with this the outcome in our outpatients was better as compared to inpatients. A longer duration of illness prior to treatment seems to be unfavourable [17,22,25]. With regard to dissociative disorder subgroup an earlier study reported a relatively worse outcome in pseudoseizure patients [20]. In contrast more recent findings pointed to a favourable course over a six year period with recovery rates of 82% and a mean symptom survival time following treatment of 1.5 years [24]. In our sample pseudoseizure patients did not have a more serious course. Polysymptomatic dissociative symptoms were associated with a less favourable course in our and another study [58]. In pseudoseizure patients those with more seizure types improved less [24]. However, Goodyer & Mitchell [20] did not find an association between number of dissociative symptoms and outcome. Co-morbidity was in part related to worse outcome [2,25]. We also found a tendency for a lower adaptation for patients with co-morbid anxiety.

## Limitations

We reinvestigated a clinical sample of child psychiatric patients. Therefore study results only hold for patients being referred to psychiatric care and cannot be generalized to subjects suffering from dissociative disorders in the community. Within our retrospective design variables in childhood had been recoded using the patients' case charts. This might be associated with a rather low reliability as compared to prospective studies. There is a need for studies on the long-term course using a prospective design. The high rate of non-participation in our study was disappointing. Apart from childhood variables not associated with non-participation in our sample there may be undiscovered aspects of the clinical course related to participation and non-participation in the follow-up investigation. However, character and extension of possible influences remain unknown. Our sample size is rather small, which goes along with a lack of statistical power. This may account for some of the null findings. Particularly with regard to prognostic features our results remain preliminary.

## Conclusion

In contrast to a rather favourable initial treatment outcome long-term course of juvenile dissociative disorder proves to be more serious with respect to psychiatric morbidity in adulthood. Treatment strategies have to consider that in a significant portion of young patients initial recovery may not be stable over time. Even after stabilisation low frequent contacts can be recommended to allow for contingent intervention in the case of recurrence of dissociative symptoms or other psychopathological states. Hereby, the development of somatization, anxiety and depressive symptoms should also be taken into account. Our knowledge on the development of juvenile dissociative disorder and on predictors of the clinical course is sparse. There is a need for further studies on the long-term course of the disorder using a more thorough design (large N, prospective, quasi-experimental).

## Competing interests

The authors declare that they have no competing interests.

## Authors' contributions

All named authors have contributed substantially to the scientific process leading up to the writing of the paper and are entirely responsible for the scientific content of it: TJ participated in the design and organization of the study, performed parts of the statistical analysis and drafted the manuscript. SS–S and TW participated in the design, organization and data collection of the study and performed the statistical analysis. AW, CW, HE, and WS participated in the design and coordination of the study and helped to draft the manuscript. All authors read and approved the final manuscript.
